# Distinct Optical Chemistry of Dissolved Organic Matter in Urban Pond Ecosystems

**DOI:** 10.1371/journal.pone.0080334

**Published:** 2013-11-07

**Authors:** Nicola A. McEnroe, Clayton J. Williams, Marguerite A. Xenopoulos, Petr Porcal, Paul C. Frost

**Affiliations:** 1 Department of Biology, Trent University, Peterborough, Ontario, Canada; 2 Biology Centre of the Academy of Science of the Czech Republic, v.v.i., Institute of Hydrobiology, České Budějovice, Czech Republic; University of Yamanashi, Japan

## Abstract

Urbanization has the potential to dramatically alter the biogeochemistry of receiving freshwater ecosystems. We examined the optical chemistry of dissolved organic matter (DOM) in forty-five urban ponds across southern Ontario, Canada to examine whether optical characteristics in these relatively new ecosystems are distinct from other freshwater systems. Dissolved organic carbon (DOC) concentrations ranged from 2 to 16 mg C L^-1^ across the ponds with an average value of 5.3 mg C L^-1^. Excitation-emission matrix (EEM) spectroscopy and parallel factor analysis (PARAFAC) modelling showed urban pond DOM to be characterized by microbial-like and, less importantly, by terrestrial derived humic-like components. The relatively transparent, non-humic DOM in urban ponds was more similar to that found in open water, lake ecosystems than to rivers or wetlands. After irradiation equivalent to 1.7 days of natural solar radiation, DOC concentrations, on average, decreased by 38% and UV absorbance decreased by 25%. Irradiation decreased the relative abundances of terrestrial humic-like components and increased protein-like aspects of the DOM pool. These findings suggest that high internal production and/or prolonged exposure to sunlight exerts a distinct and significant influence on the chemistry of urban pond DOM, which likely reduces its chemical similarity with upstream sources. These properties of urban pond DOM may alter its biogeochemical role in these relatively novel aquatic ecosystems.

## Introduction

In the last few decades, urbanization has been associated with widespread loss of natural (wetlands and forest) and agricultural areas [1]. This landscape conversion has been accompanied by greater imperviousness of watersheds to infiltration by surface waters and altered hydrological cycles (e.g., [2,3]). To mitigate flash flooding created by rapid drainage from highly impervious surfaces, newer urban landscapes often contain relatively shallow ponds (depth of ~2 m or less). While primarily built to retain and slow the downstream movement of stormwater, these ecosystems also potentially improve water quality through the retention of suspended sediments and dissolved nutrients [4]. Urban ponds are also thought to provide important ecological function by increasing biodiversity, serving as wildlife habitat, and altering biogeochemical cycles [5,6]. 

The biogeochemical role (especially for elements other than phosphorus) that urban ponds play in the developed landscape has not been well-studied, despite potential management implications. One study based on 26 urban stormwater ponds reported high rates of microbial activity and biogeochemical processes suggesting that, at current pond density, they could play an important role in regional and global carbon (C) cycles [7]. Other small, shallow freshwater systems are also typically characterized by disproportionately high rates of nutrient processing compared to larger bodies of water [8]. For example, sedimentation rates and burial of organic C can be higher in small freshwater bodies than in larger aquatic ecosystems [9-11]. In addition, allochthonous derived DOM has been shown to fuel surprising quantities of microbial production in small aquatic ecosystems and to support freshwater food webs beyond that provided by primary production alone (e.g., [12-15]). 

While not particularly well-studied, urban land use has been reported to increase the loading of dissolved organic matter (DOM) above levels found in natural areas due to changes in soil and drainage conditions [16]. In contrast, DOM concentrations in streams have been observed to decrease post-urbanisation due to the loss of hydrological flow paths through shallow soils [17,18]. Either way, DOM characteristics from such areas may differ from that seen in waters emerging from more natural (e.g., forested and wetland) or agricultural areas [19,20]. For example, DOM derived from urban wastewater can be distinguished from the DOM in receiving river systems through point-source increases in protein-like DOM resolved by fluorescence characterization [21]. DOM arising within urban aquatic ecosystems may have different transparency, humicity, molecular weight, and/or photoreactivity arising from limited contact with organic soils and vegetation in urban watersheds. If so, exported DOM from these urban environments could be less colored, more transparent, and/or have other, distinct fluorescent properties. 

The source and chemical properties of urban pond DOM could affect the rate of DOM microbial decomposition and photodegradation. Autochthonous DOM tends to be more aliphatic and less aromatic in character compared to terrestrial derived DOM (e.g., [22]). These properties have been found to limit DOM photoreactivity (e.g., [23-25]). Photodegradation can result in more biologically available substrates (e.g., [26]) or could increase DOM humicity, reducing its bioavailability (e.g., [24,27-30]). However, how DOM in these urban ponds changes in response to exposure to solar radiation remains unknown.

In this study, we first examined the quantity and quality of DOM in urban ponds as it compares to DOM sampled from more natural aquatic ecosystems. We then examined the photochemical reactivity of urban pond DOM. Finally, we examined how the properties of DOM relate to indices of internal production (e.g., seston chlorophyll). We expected that urban pond DOM would have a distinct optical chemistry compared to that derived from more natural areas, based on either greater contributions of anthropogenic derived organic matter or from high autochthonous production. We further expected that these unique chemical properties would affect rates of *in-situ* photochemical degradation. We thus provide a thorough examination of DOM and its chemistry in stormwater retention ponds. 

## Methods

### Ethics Statement

All field sites were accessed by public right of way and did not require explicit permission from the municipality to collect water. Because we only collected water samples, none of our field work involved threatened or endangered species.

### Sample sites

We examined DOM properties in forty-five urban ponds in four municipalities in southern Ontario, Canada (Ottawa, Peterborough, Richmond Hill and Whitby). Selected ponds were all in residential areas and have variable catchment and drainage properties (Table 1). Additional information about these ponds and their biogeochemistry is provided in [7,31]. We compared our DOM data on urban pond water with previously published data that we compiled from studies of non-urban, aquatic environments to determine whether their DOM characteristics match that seen in natural ecosystems. Comparison data were taken from studies of different types of aquatic environments including forested and wetland-dominated streams, saline lakes, and eutrophic to hypereutrophic inland water bodies.

**Table 1:** Selected characteristics of urban ponds in this study.

**Pond Name**	**Municipality**	**Year built**	**Land-use**	**BSD (m)**	**A_0_ (m^2^)**	**% I**
***Water St.***	***Peterborough***	***1977***	***R***	***0.75***	***3374***	***44***
***Carnegie***	***Peterborough***	***2005***	***R***	***1.5***	***5171***	***52***
***Chemong***	***Peterborough***	***2000***	***R***	***1.6***	***1063***	***64***
***Glenforest***	***Peterborough***	***1975***	***R***	***1.8***	***1657***	***61***
***Ravenwood***	***Peterborough***	***2000***	***R***	***1.5***		***64***
Tobin Court	Peterborough		R	0.6		
***White***	***Peterborough***	***2007***	***R***	***1.5***		***55***
***Loggerhead***	***Peterborough***	***2007***	***R***	***1.1***	***5164.6***	***42.6***
***Foxmeadow***	***Peterborough***	***2003***	***R***	***0.5***		***46.8***
7-3	Richmond Hill	2002	R	1.2	1027	45
7-4	Richmond Hill	1998	R	0.8	2030	44.9
***8-3***	***Richmond Hill***	***1999***	***R***	***2.76***	***1698***	***44.9***
***2-3***	***Richmond Hill***	***2000***	***R***	***1.45***	***1688***	***35.1***
9-9	Richmond Hill	2004	R	1.65	4314	
***16-8***	***Richmond Hill***	***1996***	***R***	***0.95***	***951***	***39.3***
***16-4***	***Richmond Hill***		***R***	***1***	***4620***	
***17-3***	***Richmond Hill***	***1987***	***R***	***0.75***	***7500***	***45***
***19-9***	***Richmond Hill***	***2001***	***R***	***1.5***	***2098***	***41***
***19-8***	***Richmond Hill***	***1997***	***R***	***1.5***	***1549***	***44.4***
***19-4***	***Richmond Hill***	***1997***	***R***	***2.1***	***3120***	
***9-5***	***Richmond Hill***	***2000***	***R***	***1***	***1129***	***36***
***9-6***	***Richmond Hill***	***2000***	***R***	***1***	***1698***	***50***
***8-10***	***Richmond Hill***	***2004***	***R***	***1.1***		***50***
***57-01***	***Whitby***		***R***	***1.8***		
***65-01***	***Whitby***	***2001***	***R***	***2.0***		
***68-02***	***Whitby***	***2000***	***R***	***2.6***		
***33-01***	***Whitby***	***1996***	***R***	***0.9***		
***34-02***	***Whitby***	***1995***	***R***	***0.7***		
***03-02***	***Whitby***	***1999***	***R***	***0.7***		
SWF-1409	Ottawa	1999	R	1.15	21000	10.3
SWF-1410	Ottawa	1998	R	1.3	29000	13.16
SWF-1139	Ottawa	2004	M	1.45	1000	29.94
SWF-1206	Ottawa	1999	M	1.02	10000	19.82
SWF-1207	Ottawa	1995	R	1.65	11000	33.86
SWF-1211	Ottawa	1980	R	2.1	22000	42.25
SWF-1215	Ottawa	1980	R	1.51	4000	46.57
SWF-1227	Ottawa	2000	R	1.04	11000	12.35
SWF-1306	Ottawa	2000	R	1.8	5000	30.29
SWF-1309	Ottawa	2000	R	0.9	6000	16.39
SWF-1320	Ottawa	1990	R	1.15	1000	43.47
SWF-1610	Ottawa	1979	R	1.35	1000	39.55
SWF-1611	Ottawa	1980	M	1.13	3000	
SWF-1628	Ottawa	2000	M	0.65	300	40.93
SWF-1902	Ottawa	1987	I	0.5	1000	17.5
SWF-1930	Ottawa	2000	R	1.84	33000	31.31

Ponds denoted by bolded italics were included in the photo-irradiation experiment (n=25). Data provided courtesy of each municipality when they were available.

Landuse=R(residential), I(industrial), M(mixed), H(highway); BSS= Bottom sampling depth; A_0_=Pond surface area; %I=impervious surface.

### Ambient DOM characteristics

We sampled water in ponds twice (June and August) during the summer of 2009. Whole water samples were collected at the deepest part of each pond at the surface (0-10 cm) and at the water-sediment interface (~0.5-2.7 m, Table 1) using a Van Dorn sampler. These samples were placed into deionized water (DI) rinsed, acid washed polypropylene bottles and held on ice in the dark during transport back to the laboratory. Within 24 hours, we filtered samples (Whatman Type polycarbonate PCTE, 0.2 µm) and placed this water into acid washed and pre-combusted glass amber bottles, which were refrigerated at 4°C until further analysis. Concentrations of DOC (mg C L^-1^) in each sample were determined using a TOC-TN Analyser (Model 1030D OI Analytical Aurora, Texas, USA) after combustion (750°C) and acidification with 2 N hydrochloric acid. UV-visible absorbance were measured on each sample between 200 and 800 nm (Perkin Elmer Lambda 25 Spectrophotometer) and the absorption coefficient (a_nm_) was determined by multiplying absorbance (A_nm_) by 2.303 and dividing by the path length in meters following [32]. Molar absorptivity at 280 and 350 nm (ε_280_, ε_350_) was calculated as absorbance (A_nm_) at 280 and 350 nm divided by the DOC concentration (µmol C L^-1^; [33]). Absorbance at 440 nm (A_440_) was used as an additional indicator of DOC color [34].

### DOM optical characteristics

To determine DOM fluorescence characteristics of urban pond water, we selected a subset of ponds from August sampling (n=25, Table 1) for excitation emission matrix (EEM) measurements. EEM measurements on surface water samples from these ponds were made using fluorescence spectroscopy (Varian Eclipse Fluorometer, 5 nm bandwidth, integration time 0.25 s), over a range of emission (270-600 nm, 2 nm intervals) and excitation (230-500 nm, 5 nm intervals) wavelengths. The EEMs were corrected for inner filter effects [22,35], and for second-order Raman and Rayleigh scatter effects and instrument bias using manufacturer recommended instrument settings. Milli-Q water blank EEM fluorescence was subtracted from that of sample EEMs and EEMs were converted to Raman Units (RU) using the area under the Milli-Q Raman scatter peak at excitation 350 nm. 

**Figure 1:**
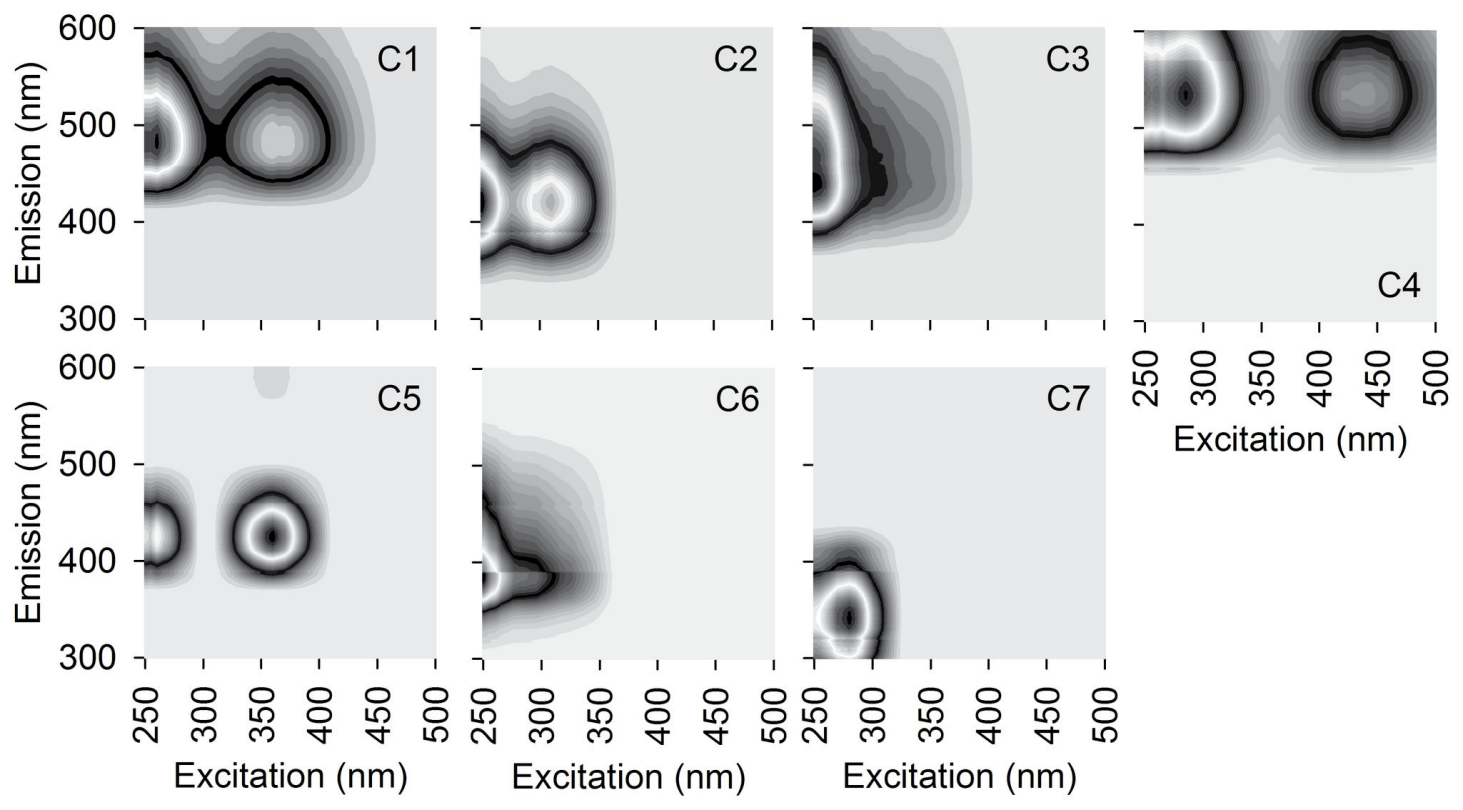
Countor plots of seven component PARAFAC model.

To investigate DOM fluorescence parameters, EEM data were used to calculate the humification index (HIX), fluorescence index (FI) and β:α ratio. The HIX was calculated as the ratio of peak area under each curve at emission 434-480 nm and 300-346 nm after excitation at 255 nm [36]. The FI was calculated, using an excitation wavelength of 370 nm, as the ratio of emission intensities at 470 and 520 nm after [22], and β:α ratio was calculated, using an excitation wavelength of 310 nm, from emission intensity at 380 nm (β region), divided by the emission intensity maximum observed between 420 and 435 nm (α region; [20,37]). A spectral slope ratio (Sr; [38]) was determined as the ratio of the log-transformed slope between 275 to 295 and 350 to 400 nm,

A seven component PARAFAC (Parallel Factor Analysis) model was used to examine factors of each EEM (Matlab 2012b, Mathworks; Figure 1). EEMs collected during the photo-irradiation experiment were fit to the PARAFAC model and residuals were visually examined to verify model fit. The full model validation and description is presented in detail in [7]. Of the seven PARAFAC components, C1 resembles humic-like material and occurs in most ecosystems [39,40]. C2 and C3 appear similar to humic-like DOM of terrestrial origin [41]. C4 resembles the fluorescence signatures of soil, fulvic acid-like substances [42]. C5 and C6 are similar to microbial derived humic-like substances [7,19,39]. C7 resembles protein-like materials [21,42] as well as photodegradable plant tannin-protein complexes [43]. The PARAFAC components are expressed as relative abundance (% of F_max_). 

### DOM photo-irradiation

The same samples (n=25) collected from the pond surface were included in photo-irradiation experiments. For these experiments, aliquots (25 ml) of filtered pond water were transferred into DI-water rinsed, sterile UV-transparent polyethylene bags and the samples were irradiated using a XE Arc lamp (500 W m^2^ for 12 hours; Suntest XLS+ Solar Simulator, 2220W lamp, internal water bath temperature of 25°C, Atlas, Germany) approximating the solar spectral quality at sea level. Control samples treated as irradiated samples (membrane filtration and stored in polyethylene bags) were wrapped in aluminium foil and exposed to the same temperature as irradiated samples inside the solar simulator. The radiation intensity of 500 W m^-2^ is equivalent to a radiant energy dosage of 21.6 MJ m^-2^, or to 1.7 days of natural solar radiation in June at our study location, based on estimates of the monthly averaged insolation on a horizontal surface (NASA Surface Meteorology and Solar Energy data; http://eosweb.larc.nasa.gov). The transformation of cumulative irradiation energy to the number of corresponding days of natural irradiation was done by dividing the cumulative energy by averaged insolation energy at the sampling location. Changes in DOC concentrations during photo-irradiation are expressed against cumulative irradiation energy, which is the product of intensity of irradiation and time. The decrease in DOC with cumulative irradiation energy followed exponential decrease as observed in other studies (e.g. [44,45]). Thus the pseudo first order kinetics rate constant was used as a measure of DOC photodegradation in individual samples, following:


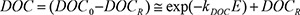
(1)

Where DOC_0_ is the initial DOC concentration, DOC_R_ is the concentration of DOC that remained after the experiment, *k*_DOC_ is the photochemical degradation rate constant (m^2^ MJ^-1^) and E is the cumulative energy of irradiation (MJ m^-2^; see 45). 

### Indices of production in urban ponds

We examined how urban pond DOM properties related to indices of production: seston chlorophyll, dissolved oxygen (DO) and total phosphorus (TP) concentrations. Seston chlorophyll concentrations on filtered samples (Whatman Type GF/F, 25 mm) were determined after ethanol cold extraction (24 h) with fluorometry (440 nm excitation, 660 nm emission wavelengths). Profiles of water column DO were obtained from the deepest part of each pond from the surface and every 10-20 cm depth to the water sediment interface (YSI Model 54 Oxygen Meter, Yellow Springs Instrument Co., Yellow Springs, Ohio, USA). TP concentrations were determined on unfiltered water samples stored at 4°C in acid washed polypropylene and pre-combusted (500°C) amber glass bottles, using potassium persulfate digestion (autoclave at 121°C) followed by ascorbic acid-sodium molybdate blue colorimetric analyses (Spectrophotometer, Biochrome Ultrospec Pro 500, 885 nm wavelength). 

### Statistical analyses

Prior to statistical analysis, most data were transformed (log(y) or square root (y)) to meet the assumptions of normality and homoscedasticity. Pearson’s Correlation analyses were used to determine significant relationships (α = 0.05) between DOM UV absorbance and fluorescence indices. One-way Analysis of Variance (ANOVA) or Student's t-test was used to determine statistically significant differences between means, followed by the Tukey-Kramer HSD post hoc test where differences were found (α = 0.05). Permutational Multivariate (M)ANOVA with post hoc comparison was used to determine the overall impact of photo-irradiance on fluorescent DOM. MANOVA results were visualized using Principle Component Analysis. Statistical analyses were carried out using JMP v.9 (SAS Institute, 2009), PASW Statistics 18 (SPSS Inc., 2009), and R with the VEGAN library. 

## Results

### Ambient DOM characteristics

In the entire dataset for 45 urban ponds, including both sampling dates and depths, DOC concentrations ranged between 2.0 to 16.2 mg L^-1^ (mean ± SD; 5.3 ± 1.9 mg L^-1^). On average, DOC concentrations were lower in June (*p* = 0.04, 4.9 mg C L^-1^) compared to August (5.7 mg C L^-1^). While molar absorptivity (ε_280_) was significantly higher in June (*p* < 0.001), absorbance at 280 (a_280;_ Table 2) and 440 nm (a_440;_ Table 1) were not different between sampling dates and showed a wide range among sampled ponds.

**Table 2:** Range in DOC concentrations, molar absorptivity at 280 nm (ε_280_) and corresponding absorption coefficient (a_280_) for 45 urban ponds in June and August, 2009.

**Site**	**DOC (mg C L^-1^)**	a_280_ **(m^-1^)**	ε_280_ (**L mol C^-1^ cm^-1^**)	a_440_ **(m^-1^)**	**FI**	**HIX**	**β:α**	**Source**
**Stormwater Ponds**								
Urban ponds, southern Ontario^June^	2.0-11.3	8.9-31.5	92-428	0.3-3.4				This study
Urban ponds, southern Ontario^Aug^	3.6-16.2	12.1-29.1	86-258	0.4-1.4	1.35-1.58	4.5-7.9	0.74-0.90	This study
**Wetlands, Streams and Rivers**								
Agro-Urban Streams, Australia	2.0-140.0				1.20-1.44		0.40-0.70	[40]
43 mixed watershed streams, southern Ontario, Canada	4.1-26.4	5.5-72.5	133-369		1.19-1.47	8.7-32.2	0.48-0.70	[19]
Subtropical wetland	4.6-45.0	4.8-81.9			1.28-1.47			[50]
Okavango Delta wetland, Botswana	13.3-16.6		276-324		1.45-1.50			[58]
Harp Lake watershed, south-central Ontario	10.0-35.0				1.1-1.2			[59]
Lake Superior watershed streams	3.5-34.0		138-586					[60]
Deer Creek, CO, USA	1.3-4.1				1.40-1.48			[61]
Suwannee River, GA, USA	35		509					[62,63]
**Lakes**								
27 Prairie lakes	13.4-328					1.4-6.4		[49]
Alpine Lake	0.5-1.7				1.33-1.71			[64]
Antarctic Lakes	2.5-22.6				1.71-2.71			[61]
Antarctic Lake (Pony)					1.5	20		[61]
30 temperate lakes	3.7-21.5			0.8-19.3				[65]
Antarctic Lake (Fryxell)			150					[62]

Absorbance at 440 nm is represented as a_440_. Data is presented in comparison to other published studies.

**Figure 2:**
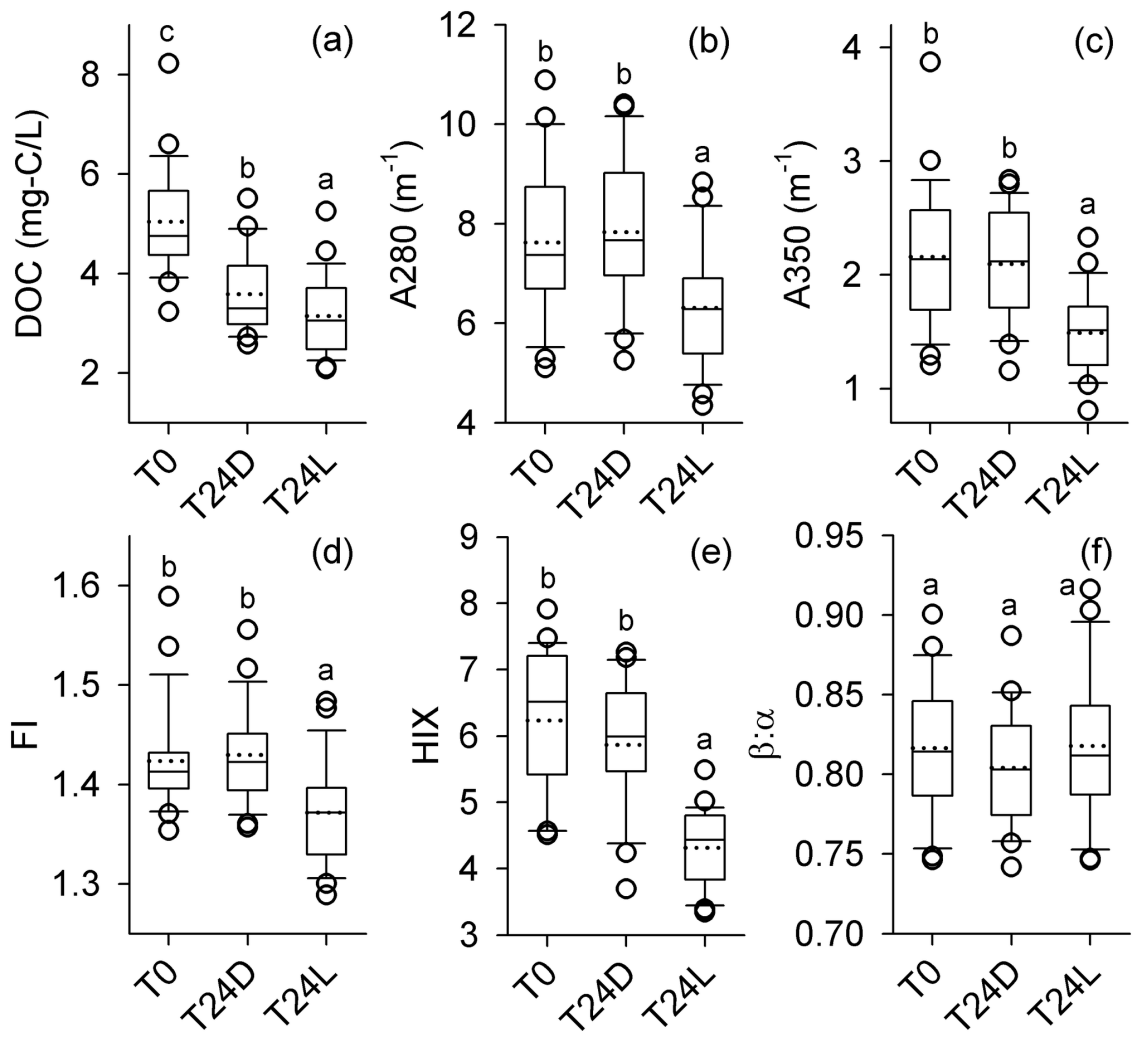
Effects of photo-irradiation on urban pond dissolved organic matter. Box plots showing the median (solid line), 25^th^ and 75^th^ percentiles (boxes) and 10^th^ and 90^th^ percentiles (dots) for absorbance (A_nm_) at (a) 280 and (b) 350 nm and for (c) DOC concentrations and measured fluorescence indices (d) FI, (e) HIX and (f) β:α for 25 urban ponds in August, prior to photo-irradiation (T0) and for light treatment (T24L), and dark treatment (T24D). Values denoted with different letters are significantly different (*p*<0.05).

**Figure 3:**
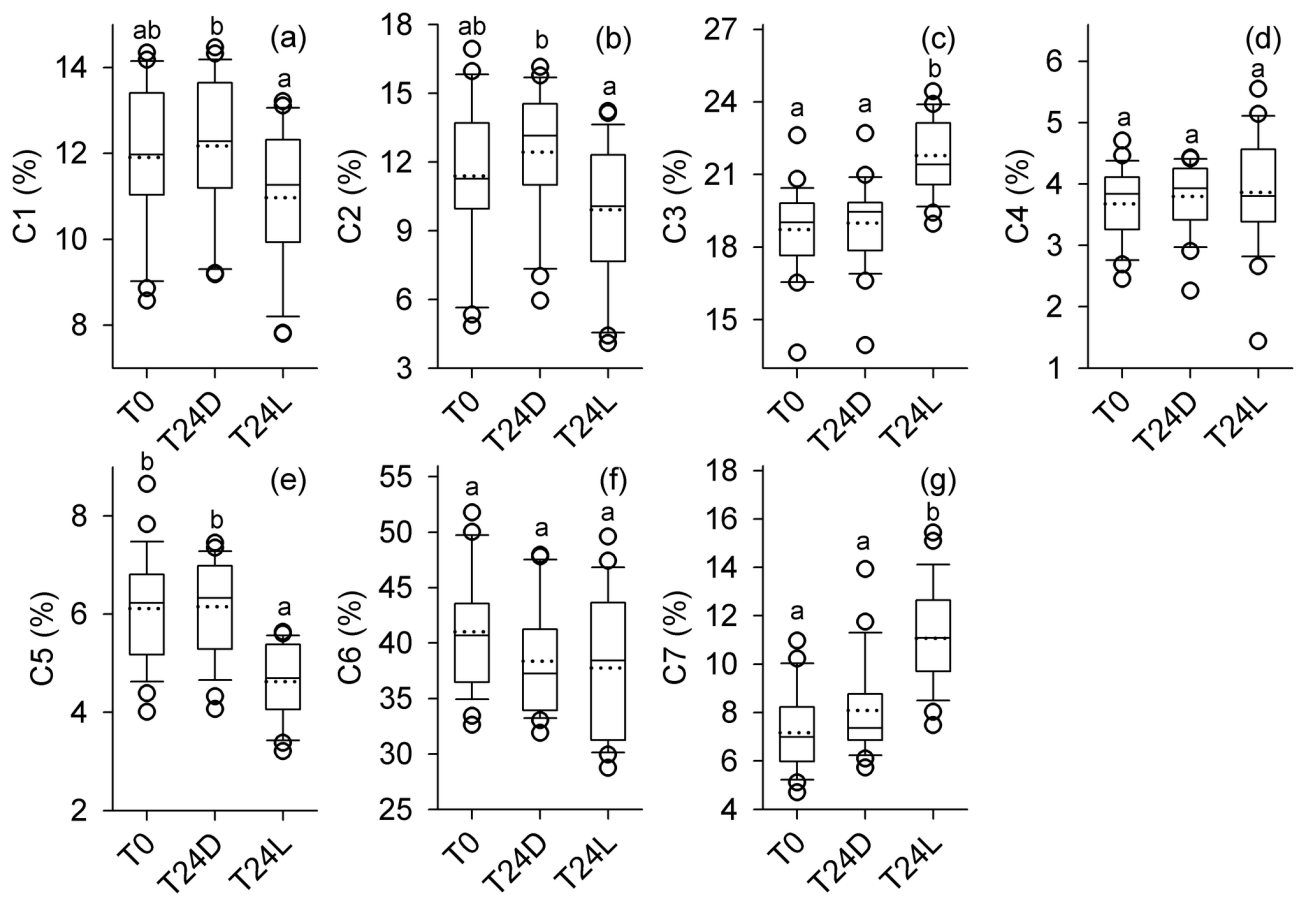
Responses of PARAFAC components (C1-7; %) to photo-irradiation of urban pond DOM. Box plots show the median (solid line), mean (dotted lines), 25^th^ and 75^th^ percentiles (boxes), 10^th^ to 90^th^ percentiles (whiskers), and >90^th^ percentiles (dots) prior to photo-irradiation (T0), after dark treatment (T24D), and after light treatment (T24L). Values denoted with different letters are significantly different (*p*<0.05).

From the subset of urban ponds selected for more detailed optical characterization (n=25), DOC concentrations ranged from 3.2 to 8.2 mg L^-1^ (mean ± SD; 5.03 ± 1.04 mg L^-1^) and fluorescence indices were found to vary considerably (Figure 2; Table 2). DOC concentrations were positively correlated with FI and UV-visible absorbance and were negatively correlated with molar absorptivity (ε_280_; Table 3). While PARAFAC component C6 (anthropogenic/microbial humic-like) was generally the most abundant (23 - 52%) and C4 (soil fulvic acid-like) was the least abundant (2 - 5%), the relative abundance of the individual components varied among ponds (Figure 3). These components were also related to other optical properties of the DOM. C6 correlated positively with β:α and negatively with ε_350_ (Table 3). C1 (terrestrial humic-like), C2 (terrestrial humic-like), and C4 correlated positively with HIX and ε_350_ but negatively with β:α (Table 3). C7 (protein-like) generally showed the opposite relationships from the humic-like components. C4 was the only PARAFAC component that correlated with DOC, indicating that urban pond fluorescence DOM quality varied among ponds mostly independent of bulk DOC concentration. Spectral slope (Sr) was significantly related (negatively) to one component, C7 (r=-0.72; p<0.001), and was not related to any other PARAFAC components (Table 3).

**Table 3:** Pearson’s correlation coefficient (r) between DOC concentrations and UV-Visible absorbance and fluorescence indices for 25 urban ponds in August, prior to photo-irradiation (T0).

	DOC	C1	C2	C3	C4	C5	C6	C7
DOC	1	ns	ns	Ns	**-0.51**	ns	ns	ns
A_280_	**0.65**	ns	ns	Ns	ns	ns	ns	ns
A_350_	*0.48*	ns	*0.44*	*-0.45*	ns	*-0.50*	ns	ns
ε_280_	*-0.42*	ns	ns	Ns	**0.62**	ns	ns	*-0.41*
ε_350_	ns	**0.52**	*0.49*	Ns	*0.49*	*0.49*	*-0.41*	ns
HIX	ns	*0.50*	*0.40*	Ns	*0.40*	ns	ns	**-0.85**
FI	**0.53**	ns	*0.45*	**-0.82**	*-0.47*	**0.72**	ns	ns
β:α	ns	**-0.62**	**-0.62**	Ns	*-0.42*	ns	**0.60**	*0.45*
Sr	ns	ns	ns	Ns	ns	ns	ns	**-0.72**

Significant correlations are indicated in bold (*p*<0.01).

### DOM photo-irradiation

In samples exposed to artificial light, pseudo-first order rate constants ranged between 0.003 and 0.03 m^2^ MJ^-1^ after a cumulative irradiation energy exposure of 21.6 MJ m^-2^ and were significantly higher than dark treatment rate constants (*p* < 0.001; range 0.0003 to 0.02 m^2^ MJ^-1^). In 20% of samples, photo-irradiation had no effect on DOC concentrations with net irradiation rate constants ranging from 0 to 0.02 m^2^ MJ^-1^. Despite this, photo-irradiation decreased DOC concentrations, on average, by 38%. We also observed decreases in absorbance (A_nm_) and fluorescence indices in samples exposed to light relative to the dark controls (*p* < 0.05; Figure 2). While average absorbance (A_nm_) was reduced by 25% in the UVA and UVB regions, average molar absorptivity (ε_280_) increased by 30% in light exposed samples relative to the dark controls. 

**Figure 4:**
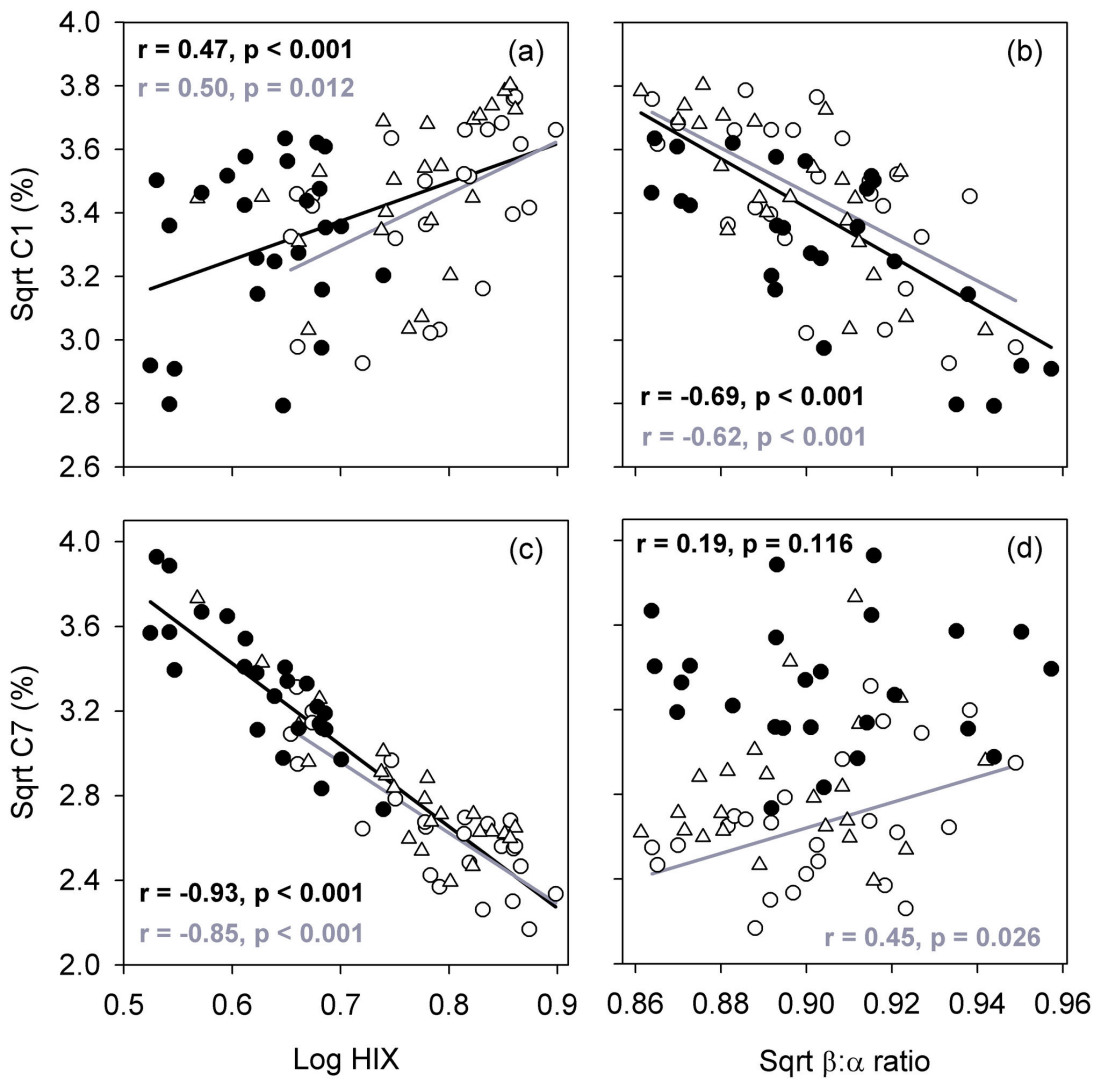
Photo-induced changes in DOM chemistry. Correlations between the EEM-PARAFAC model C1 (a,b) and C7 (c,d), and the humification index (HIX; a,c) and β:α ratio (b,d) for 25 urban ponds in August (open circles=initial (T0, prior to photo-irradiation), closed circles=light treatment (T24L), triangles=dark treatment (T24D). Correlations are presented for the overall data set (black lines) and T0 (gray lines).

**Figure 5:**
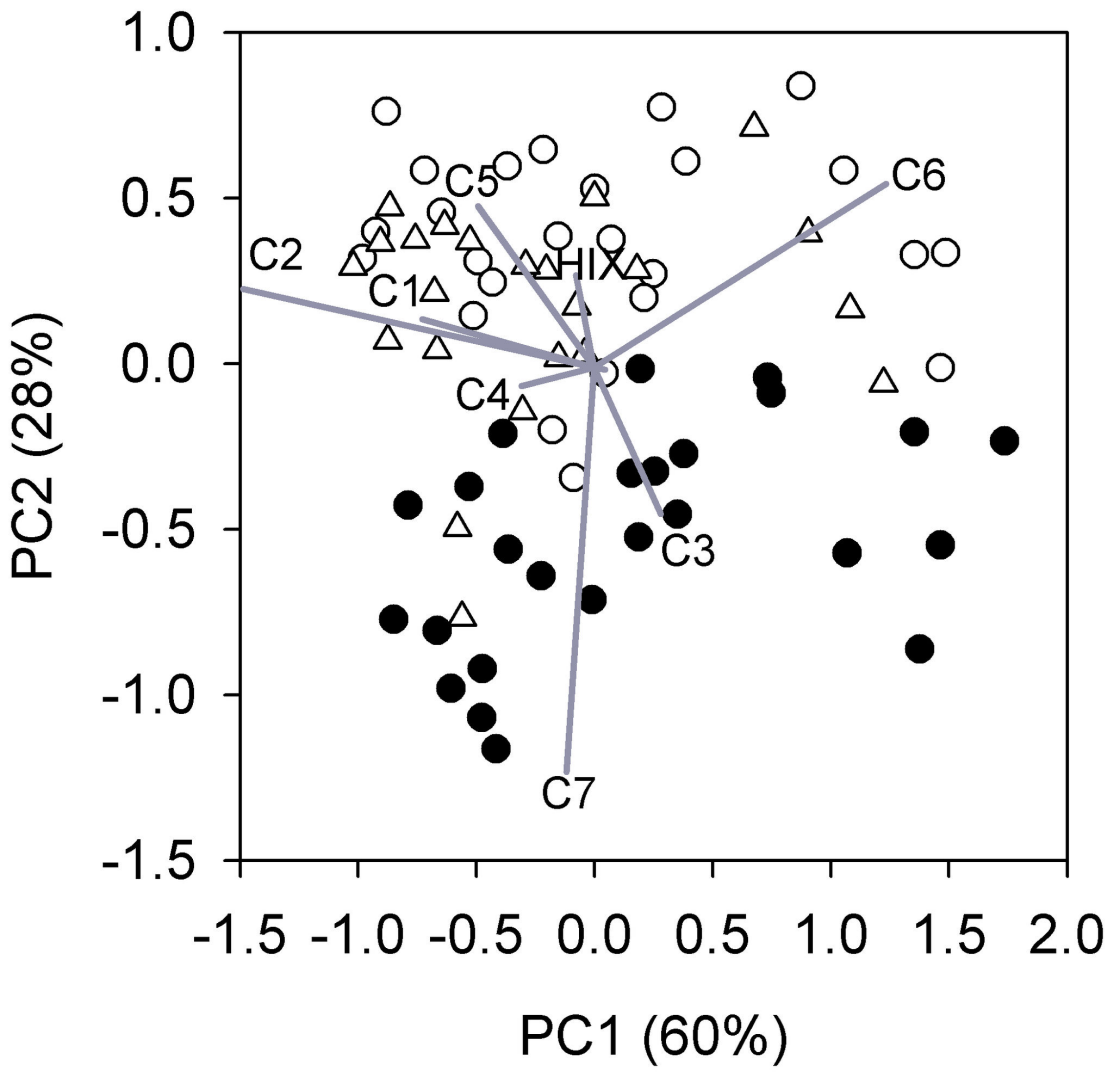
Principle components plot with vector loadings for the fluorescent DOM pool. MANOVA with post hoc test indicated that the light exposed fluorescent DOM pool (closed circles) was significantly different than initial (open circles) and dark (triangles) pools, which did not differ. Note, vector loadings for β:α and FI were near the origin in the first two PCA dimensions and are not displayed in this figure.

Photo-irradiation altered characteristics of DOM quality indexed by PARAFAC, but these effects depended on the component examined (Figure 3). Among all ponds, the intensity C1 to C6 (F_max_; RU) decreased after light exposure. The intensity of C7 (RU), however, responded non-uniformly to light exposure among ponds. C7 (RU) increased, decreased, or was similar to initial F_max_ depending on the pond water examined. The relative abundance (%) of C4 and C6 were not significantly different from initial levels after dark and light incubations (*p* = 0.753 and *p* = 0.095, respectively). C1 (%) and C2 (%) were significantly lower after light exposure relative to the dark incubation (*p* = 0.039 and *p* = 0.027, respectively). Photo-irradiation also significantly decreased C5 (%; *p* < 0.001) and increased C3 (%; *p* < 0.001) and C7 (%; *p* < 0.001) from dark incubated and initial treatments. The relative abundance of C1 consistently correlated positively with HIX and negatively with β:α across initial, light, and dark treatments (Figure 4). Correlations between C7, HIX, and β:α, however, were influenced by photo-irradiation (Figure 4). Light treated samples had higher levels of C7 and lower levels of HIX than initial and dark samples and had a similar correlation slope overall. C7 positively related to β:α for initial samples, but this relationship was not preserved after photo-irradiation. Overall, photo-irradiation significantly impacted the fluorescent DOM pool (MANOVA, *p* = 0.001), which appeared to be strongly influenced by changes in C7 (Figure 5). 

### Index of urban pond production

**Figure 6:**
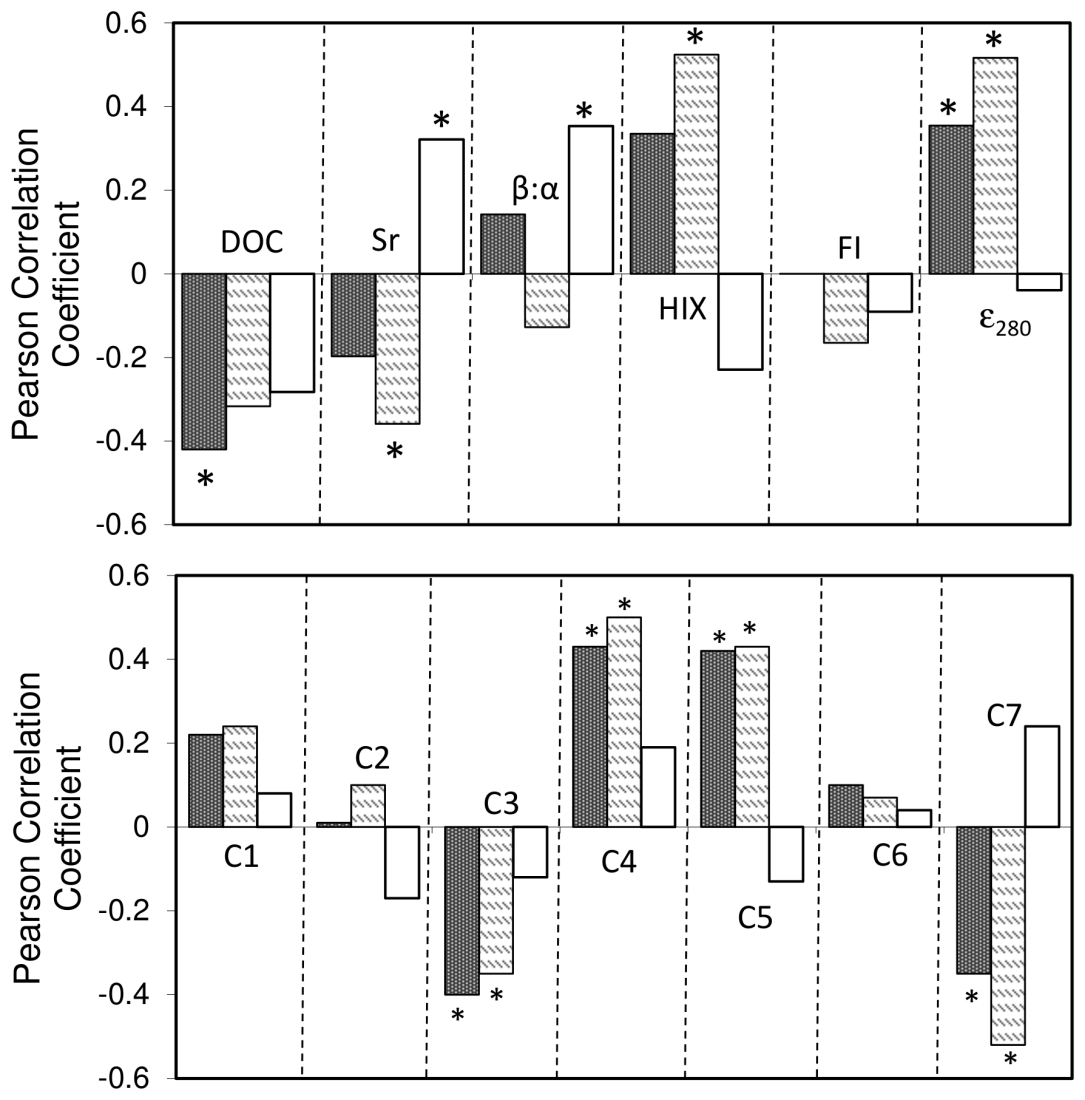
Correlation coefficients between DOC concentration (mg C L^-1^), optical properties, and PARAFAC components and seston chlorophyll (dark bars), TP (light bars) and DO (open bars). Significant correlations (*p*<0.05) are noted with *.

We found that DOC concentrations (negatively) and ε_280_ (positively) were correlated with chlorophyll whereas no other optical property of the DOM was related to this index of productivity (Figure 6). Sr (negatively), HIX (positively), and ε_280_ (positively) significantly correlated with total phosphorus. Only Sr and β:α related to dissolved oxygen concentrations in the surface waters of urban ponds (Figure 6). Of the seven PARAFAC components, we found that seston chlorophyll and total phosphorus was positively correlated with C4 and C5 and negatively correlated with C3 and C7 (Figure 6). C1, C2, and C6 were not related to any of our indices of water column productivity. 

## Discussion

We examined the quantity and chemistry of DOM in ponds embedded within highly urbanized environments and report the change in its optical chemistry after irradiation. We expected that DOM in urban ponds would have a distinct optical chemistry compared to that derived from more natural areas. This predicted difference was based on our expectations of greater contributions of either anthropogenic-derived organic matter (of a distinct composition) or from high internal production of DOM. We found that urban pond DOM was relatively transparent (low ε_280_) and uncolored (low a_440_), had lower HIX and higher β:α ratios compared to reported values for numerous temperate lakes and streams, and some other anthropogenic impacted waters. DOM characteristics from urban ponds were most similar to that found in mixotrophic to eutrophic aquatic systems where autochthonous DOM has been shown to dominate (Table 2). 

DOM in urban ponds nonetheless contained a unique mixture of microbial and terrestrial derived C, reflected in the HIX index [36], FI and β:α ratio values [39,46] (Table 1). The DOM pool was dominated by a likely algal-derived humic-like component (C6) with relatively low percentages of terrestrial-like components (Figure 3). C6 was much more abundant in urban ponds than in natural freshwaters in the same geographical region (e.g., southern Ontario [19];; C. Williams, unpublished data). It could be argued that urban ponds should not be dominated by autochthonous C, given their role in overland flood control and engineered connectivity with the watershed. The lack of terrestrial DOM influence on pond DOM may simply reflect the reduced presence of natural components (e.g. vegetation, soils, etc.) of the upstream catchment. Urban streams, however, can contain high amounts of terrestrial DOM [19,40] and we expected a stronger signal of this DOM within urban ponds. These ecosystems could either receive very different runoff or they may quickly process in-fluxing DOM. Future studies should focus on determining the relative importance of terrestrial DOM sources and internal processing rates on urban pond DOM chemistry, in part, by examining the timing and importance of external water inputs. The urban ponds in our study are downstream of stormwater drainage systems designed to channel runoff very quickly, which may further limit contact with soils and vegetation within the upstream catchment. Our results contrast with other freshwater ecosystems where allochthonous C is usually assumed to be the major fraction of DOM (e.g., [47]) due to high hydrologic connectivity between rivers and lakes and their upstream undeveloped watersheds.

The distinct character of urban pond DOM from that found in natural streams and lakes in the same region and other aquatic ecosystems largely resulted from the greater prominence of relatively transparent, internally-produced DOM. This has also been reported in other anthropogenically impacted water bodies that are relatively nutrient rich (e.g., [48] and references therein) as well as in mixotrophic to eutrophic, saline lakes (e.g., [49]). An algal-derived humic-like component (C6) was the most abundant in urban ponds and represented about 52% of the DOM fluorescence pools. C6 was higher in ponds with lower HIX scores, indicating that DOM with this chemical feature is produced internally and dilutes the allochthonous DOM pools. This component type has been documented in other human impacted areas and in highly productive ecosystems (e.g., [50]), which is further indication that there is significant internal production of DOC in urban ponds [7,41]. Urban pond DOM thus appears to have a distinct chemical signature that originates from primary production, which is fueled by high nutrient loading in these heavily urbanized catchments.

Due to its relative transparency and low aromaticity, the major DOM fraction in urban ponds should be particularly resistant to photochemical transformation. In fact, the main component of the DOM pool (C6) was not impacted significantly by light exposure. This result is similar to that reported for pelagic algal-derived DOM and DOM in some eutrophic ecosystems, which have relatively low rates of photomineralization [26]. Similarly, we also found no DOC loss with light exposure in about one fifth of our pond samples. This lack of significant total DOM photodegradation might simply reflect the chemical properties of internally produced DOM in these urban ponds. Especially transparent autochthonous material should not be photo-oxidative [25]. On the other hand, DOM in urban ponds may have already experienced prolonged UVR exposure prior to sampling, especially if there had been no recent hydrological inputs from the watershed. We would expect that rates of photochemical degradation should be higher in water samples obtained directly from in-flowing stormwater. Future comparative studies of DOM properties and photodegradation of fresh stormwater and *in situ* urban pond water would be useful to differentiate between these controls.

The less abundant terrestrial derived components of the urban pond DOM pool were the most sensitive to photochemical degradation. After photo-irradiation, DOM fluorescence shifted from humic-like to protein-like, with decreases in the most terrestrial humic-like components (C1, C2, and C5) and significant increases a protein-like component (C7; Figure 3). One terrestrial, humic-like component (C3) increased after photo-irradiation, which suggests that this component is photo-stable or photo-produced [41]. This indicates that C3 might be a product of photodegradation of the other terrestrial components. Given these changes in photo-exposed DOM samples, natural measurements of these rates of gain and losses in urban ponds over time would be a logical next step to better understand the controls of the DOM pool in these urban freshwater ecosystems. Nevertheless, DOM chemistry in urban ponds appears to be controlled by a combination of processes (i.e., DOM import, release from primary producers, and photoirradiation), which would all act in concert to shape the overall properties of this important energy source to microbial communities.

We found DOC concentration decreased in many pond samples under irradiation, but losses were also observed in some samples held in the dark. This dark-sample decrease in DOC concentration likely resulted from microbial activity [51]. While samples were filtered prior to the photo-exposure experiment, remnant microbial communities could nonetheless have consumed labile components of the DOM. The decrease in DOC concentration in dark samples was not reflected by corresponding changes in absorbance and is consistent with losses of low molecular-weight labile substrates with low absorbance. These same decreases in labile substrates with low absorbance were observed in irradiated samples, which is indicated by the increase in ε_280_ that would have been produced by a smaller decrease in absorbance compared to DOC concentration. A recent review by [52] showed variability in bacterial responses during irradiation ranging from stimulation to inhibition. However, irradiating with a full range of artificial UVA and UVB inhibited microbial growth, perhaps due to photoproduction of singlet oxygen and other reactive oxygen species [53-55]. Singlet oxygen is the primary agent of photo-oxidative stress in microorganisms [56] and high concentrations may delay consumption of the readily decomposable portions of the DOM until dark conditions prevail. Thus the microbial degradation in irradiated samples likely took place after irradiation rather than simultaneously with irradiation. The dark-sample losses nonetheless suggest that some changes observed in the photo-exposed samples resulted from the experimental conditions (i.e., non-sterility of bags) and not light per se. However, compared to the rates observed in the light exposed samples, this effect was minimal and does not significantly affect rate measurements or the observed photo-induced changes in DOM chemistry.

Chlorophyll concentrations are one surrogate index of algal biomass and should be correlated with the internal production of autochthonous DOC in freshwater ecosystems (e.g., [57]). Consequently, we expected that algal biomass in the urban ponds to be positively related to concentrations of the highly transparent DOM. Our results are not entirely consistent with this expectation as HIX and ε_280_ (both indicators of terrestrial DOM) were positively correlated with TP and chlorophyll. This result suggests that the connections between TP, productivity, and DOM chemistry are likely more complex than the simple scenario previously described. For example, greater primary production coincides with higher TP concentration in urban ponds but this may have contrasting effects on the DOM and its chemistry. While internal productivity may contribute transparent DOM and dilute terrestrial sources, it may also fuel (along with the higher nutrient concentrations) greater microbial activity that could reduce DOM concentrations. The negative relationships between chlorophyll and TP with C7 are consistent with these indirect connections. Future work should thus better examine the how DOM chemistry relates to primary producers and their external nutrient controls in urban environments.

We found a prominent signature of internally derived DOM in urban ponds of this study. This unique chemical signature likely reflects a dominance of internally produced C, the lack of external humic sources, and considerable microbial and photo-processing. While this unique chemical signature differentiates urban pond DOM from other aquatic ecosystems, how it affects the pond physical (light penetration), chemical (metal binding), or biological (microbial production) processes remains largely to be seen. Given their growing abundance and important role in urban water cycles, these ponds would appear to be potential hotspots for C processing in the urban landscape and warrant further examination of their carbon cycling.
